# Advances in lipid nanoparticles delivering genetic medicines for solid cancers

**DOI:** 10.1016/j.omtn.2026.102838

**Published:** 2026-01-15

**Authors:** Fan Yang, Tristan A. Scott

**Affiliations:** 1 Beckman Research Institute and Hematological Malignancy and Stem Cell Transplantation Institute at the City of Hope, 1500 E. Duarte Rd., Duarte, CA 91010, USA; 2 Center for Gene Therapy, City of Hope, 1500 E. Duarte Rd., Duarte, CA 91010, USA

**Keywords:** MT: Delivery Strategies, lipid nanoparticle, intratumoral, systematic, gene therapy, cancer, targeted lipid nanoparticle, moiety, passive, mRNA

## Abstract

Nucleic acid lipid nanoparticle (LNP) technology has enabled the delivery of genetic medicines to solid tumors, opening new avenues for oncology therapeutics. Solid tumors present unique challenges to nucleic acid delivery because of their complex tumor microenvironment (TME), which acts as a barrier to NP delivery. Nevertheless, nucleic acid LNPs carry diverse genetic medicine modalities that can exert anti-cancer effects. The versatility of LNPs allows both local and systemic administrations for the delivery of gene therapy payloads to solid cancer, with the additional capability to selectively target specific cell types through conjugation of targeting ligands onto the LNPs. Genetic medicines delivered by LNPs can directly affect cancer cells, such as by suppressing oncogenic drivers and cancer pathways with small non-coding RNAs, or through overexpression of toxin genes or tumor suppressors. Non-cancer components of the tumor, such as tumor-associated vasculature, can also be targeted and disrupted to inhibit the structures that support tumor growth. Alternatively, LNP delivery of genetic medicines can indirectly elicit anti-tumor effects by modifying the immune state of the tumor environment through delivery of immunomodulatory cytokines, overexpression of activatable receptors to stimulate immune cells with agonists, or antigen-binding scaffolds to direct immunity towards a cancer cell. In this review, we summarize recent advances, challenges, and prospects of LNP delivery of genetic medicines to treat solid tumors.

## Graphical abstract

**Figure:**
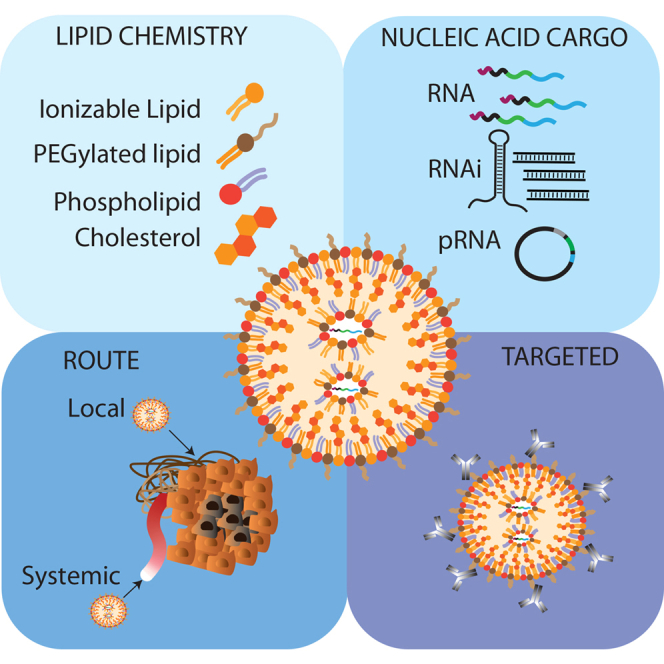

## Teaser

Lipid nanoparticle (LNP)-mediated delivery of genetic medicines to treat solid cancer has the potential to revolutionize cancer treatment. Fang and Scott review the recent advances, challenges, and prospects of LNP delivery of genetic medicines to solid cancers.

## Introduction

The success of coronavirus disease 2019 (COVID-19) mRNA vaccine supercharged the development and applications of lipid nanoparticles (LNPs) for the delivery of genetic medicines. The prior success of LNP-mediated delivery of small regulatory RNAs to treat liver-associated hereditary transthyretin amyloidosis (hATTR), later approved by the Food and Drug Administration (FDA) as the drug Onpattro, demonstrated its utility in disease-modifying activity with low toxicity.^1^^,^^2^^,^^3^ LNP-based vaccines for cancer long proceeded the momentum generated by the rapid deployment and widespread success of the COVID-19 vaccine, which further highlighted that transient, non-viral LNPs represent a robust delivery system that could be coupled with genetic medicines as next-generation solid cancer interventions.

Cancer is a complex ecosystem of cancerous and non-cancerous cells with a complex array of cellular interactions dictating disease outcomes. Broadly cancer is categorized into solid and hematological malignancies and is the result of a concert of genetic mutations and structural genomic changes induced by various stresses that form neoplastic lesions (smoking, alcohol, UV exposure, carcinogens, chronic inflammation). The accumulation of these genomic changes with oncogenic potential is then combined with cellular interactions that facilitate uncontrolled tumor growth. Solid cancers emerge as a mass of cells within a specific tissue or organ and present unique treatment challenges. For example, chimeric antigen receptor (CAR) technology, in which immune cells (traditionally cytotoxic T cells) are modified to artificially express an anti-cancer ligand attached to a signaling domain, is highly effective at inducing curative states in certain hematological malignancies.^3^ However, although significant advances have been made, the application of CAR therapy against solid cancers has faced significant delays and challenges, largely as a result of the complex and hostile tumor microenvironment (TME).^4^

**Figure 1:**
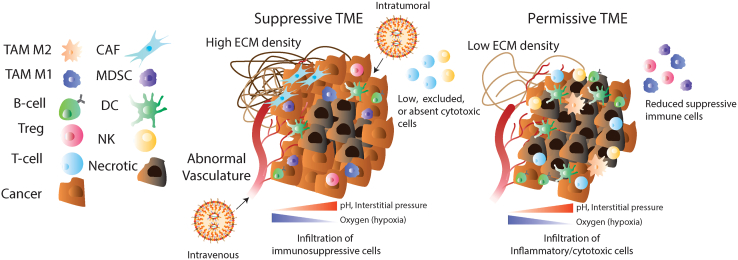
Solid tumor and tumor microenvironment Schematic of the TME in solid cancers. Various components can influence LNP delivery, including abnormal “leaky” vasculature, which can allow passive uptake of LNPs from the circulation. LNPs may be administered systemically (intravenously), entering the tumor via its blood supply, or locally (intratumorally) through direct injection into the lesion. Solid tumors often exhibit a hypoxic center with a necrotic core of dead cells, accompanied by gradients of acidity (low pH) and elevated interstitial pressure. These features can hinder LNP penetration but may also be exploited for targeted delivery (i.e., pH-sensitive lipids). The TME can be either suppressive, characterized by immunosuppressive cells and associated with poor outcomes, or immune-permissive, with increased cytotoxic and pro-inflammatory cells. A dense extracellular matrix (ECM), common in aggressive tumors, further limits LNP uptake. Key cellular components include tumor-associated macrophages (TAMs), regulatory T cells (Tregs), cancer-associated fibroblasts (CAFs), myeloid-derived suppressor cells (MDSCs), dendritic cells (DCs), and natural killer (NK) cells. Left: suppressive TME; right: permissive TME.

The TME is a multifaceted ecosystem, with cancerous cells surrounded by a non-malignant stroma embedded in a vascularized extracellular matrix (EMC).^5^ (Figure 1) The TME contains cancer cells that interact with an array of diverse immune cells, including lymphocytes (T cells, B cells, natural killer [NK] cells) and myeloid cells (macrophages, dendritic cells [DCs]), as well as other non-immune stromal cells such as fibroblasts, endothelial cells, and others.^5^ These cells may have pro- or anti-cancer effects depending on the cancer and immune context. Although cancer cells have traditionally been the target of therapeutic inventions, new insights into the roles of immune and stromal components have offered additional mechanisms for disease intervention. The TME of solid cancers can differ significantly based on tumor stage, the patient’s background, and tissue context. Apart from the TME being locally dynamic, during the process of metastasis cancer cells traverse out of the solid tumor structure and enter the bloodstream.^5^ The colonization and tumor formation at distant “pre-conditioned” organ sites presents new challenges for NP delivery, not only due to the need for delivery to multiple cancer foci in advance disease, but also because of the new tissue anatomy, which may require alternative NPs or even alternative genetic medicines.

For LNP delivery of genetic medicines to solid tumors, there are features of the TME that favor and disfavor NP delivery. In favor of NP delivery is the abnormal vasculature. Tumor cells have high metabolic rates and require sufficient nutrients and oxygen to support growth. As a result, hypoxia is a common feature of the TME, which stimulates angiogenesis, resulting in aberrant vessel structures.^6^ This vasculature is defined as “leaky” and may allow permeability to NPs; however, this concept has been recently challenged in favor of more active mechanisms of NP import into tumors, potentially offering new opportunities to improve LNP tumor delivery.^7^ (see “a new dimension in LNP delivery”).

Therapeutic free proteins that readily diffuse out of the TME reduces their effectiveness within the tumor, which has required further engineering for improved tumor retention.^8^ However, the intrinsic retention of LNPs in tumors concentrates the drug’s effect.^9^ There is heterogeneous distribution of NPs within tumors after both local and systemic administration, but other factors may allow for the spread of drugs within the tumor environment.^10^ Tumor-associated macrophages (TAMs) may play a role in the accumulation and distribution of NPs in the TME for small-molecule drug delivery, favoring deeper tumor penetration and distribution,^11^ but how this favors large biomolecules such as genetic medicines has yet to be determined. Additionally, the chemistry of LNPs can be made responsive to the TME, such as to pH or oxygen levels, to favor the local release of therapeutics within the TME and reduce systemic toxicity.

The dynamic and complex nature of the TME raises challenges for NP delivery of genetic medicines. The immune environment can sequester NPs in non-target cells, reducing delivery to the intended cell type. Furthermore, the presence of rigid and dense ECMs (collagen, fibronectin, and hyaluronan) and a “fibrotic shell” (fibroblast cells creating a barrier between the blood vessel and TME) may negatively affect the delivery of systemically administered NPs.^12^ (Figure 1) Nevertheless, significance advances have been made using LNPs to deliver genetic medicines to solid cancers as next-generation treatments. This review will cover current applications of genetic medicines delivered using LNPs to solid cancers, their indications, challenges, and possible future technologies and developments that could transform solid cancer treatment.

### LNPs for delivery of genetic medicines

Genetic medicines generally require delivery vehicles due to the presence of nucleases in the blood and interstitial spaces that rapidly degrade free nucleic acids. Once taken up by the intended tumor cell, the genetic material requires assistance to be released into the cytoplasm at functional levels to elicit a therapeutic effect. Furthermore, the delivery vehicle can be modified with targeting ligands to enable directed delivery of the genetic medicine to an intended tissue or cell. NPs shield sensitive genetic cargo from biological components that would otherwise degrade the genetic payloads or prevent cellular release.

LNPs is an umbrella term for a wide range of synthetic lipid-based nano-formulations. Traditionally, liposomes have been used for small-molecule drug delivery, as these bilayer particles can dissolve drugs in their aqueous cores and successfully deliver chemotherapeutic agents to solid tumors.^13^ However, negatively charged small nucleic acids (DNA and RNA) require other polycations to neutralize their charge for efficient loading into the aqueous core.^14^ The permanently charged lipids were toxic, had low circulation times, and interacted with anionic biomolecules, which reduced the applicability of liposomes. These limitations required further LNP modifications and innovations, which have been covered extensively elsewhere.^14^ Briefly, the addition of polyethylene glycol (PEG) molecules onto the surface of the particle (PEGylation) shielded the cationic surface, and “stealth” LNPs improved systemic distribution and circulation times.^15^ The development of the ionizable lipid transformed nucleic acid delivery, allowing the translation of the first LNP-delivered genetic medicines into the clinic and the production of a commercial drug.^16^ The ionizable lipid in advanced lipid-nucleic acid NPs has a responsive electrostatic charge based on the lipid pKa and the environmental pH. Being positively charged at low pH during production of LNPs (i.e., formulated in citric acid) allows nucleic acid binding and loading into the NP, which is then returned to physiological conditions (pH 7.4). The neutral pH neutralizes the cationic charge and prevents the sequestration of particles by scavenger cells (Kupffer cells and splenic macrophages), which typically bind positively charged particles. After the LNP reaches the target cells, it can be internalized by micropinocytosis, clathrin-mediated endocytosis, or caveolae-mediated endocytosis.^17^ When the LNP enters the cell and the acidified endosome, the low pH reestablishes a positively charged lipid to destabilize the endosomal membrane and promote cytosolic release of the nucleic acid, where, again, the neutral pH of the cytosol assists release of the negatively charged genetic cargo from the lipid and allows its subsequent interaction with the cellular machinery. For the review, we will focus on advanced ionizable lipid-nucleic acid NPs with genetic medicines.

### Lipid-nucleic acid NPs

**Figure 2:**
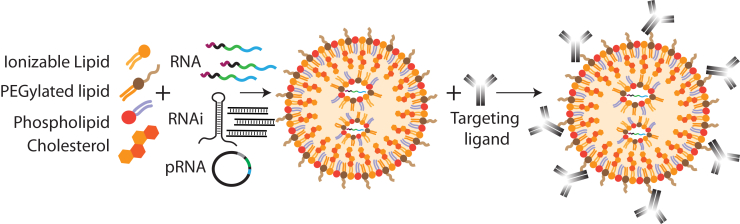
LNP for passive and active targeting in solid cancer A five-component LNP typically consists of polyethylene glycol (PEG), cholesterol, phospholipids (“helper” lipids), ionizable lipids, and a genetic cargo such as DNA or RNA. “Active” targeted LNPs incorporate a conjugated ligand on their surface, such as an antibody, single-chain variable fragment (scFv), nanobody, aptamer, or peptide, that directs the nanoparticle to its cognate receptor, enabling selective delivery.

Generally, the anatomy of LNPs consists of a five-component system with four lipids: a PEG derivative, cholesterol, a phospholipid (“helper” lipid), an ionizable lipid, and a DNA and/or RNA cargo. (Figure 2) Non-viral LNPs are comparatively less immunogenic, with favorable engineering and design features entrenched in the available chemistry and materials science. Ideally, delivery vectors should be biocompatible, allow delivery of large payloads, and have circulation times after systemic administration that are sufficient to allow accumulation and release of the genetic cargo at the tumor site. Formulations can be modified by changing the percentage ratio of lipids and the length of the PEG chains to alter circulation times and the biodistribution profile.^18^^,^^19^^,^^20^ An additional key engineerable feature that impacts LNP delivery is the ionizable lipid, which itself can affect particle size, the overall charge of the particles, efficiency of cargo encapsulation and release, and the biodistribution profile.

### Genetic medicines in NPs for solid cancer

**Figure 3:**
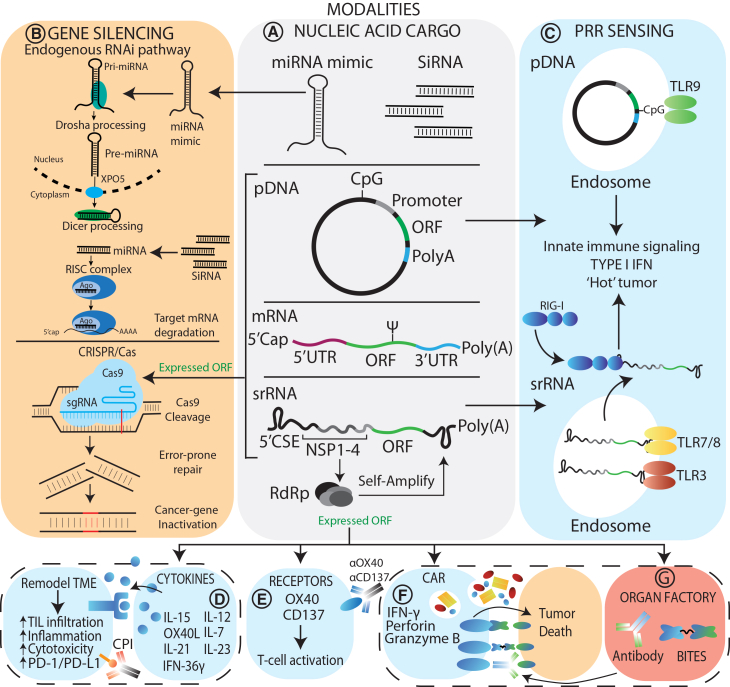
Genetic medicine with LNPs in solid cancer (A) Multiple modalities have been delivered to solid cancers using LNPs with diverse nucleic acid cargos. These include small non-coding RNAs, such as miRNAs and siRNAs, or encoding nucleic acids, such as pDNA, mRNA, or srRNA, to express anti-cancer transgenes. (B) **Gene Silencing:** Tumor cells (brown) can be targeted to elicit anti-tumor effects through suppression or inactivation of cancer-promoting genes. *Endogenous RNAi pathways:* RNAi effectors, such as miRNA mimics or siRNAs, trigger the endogenous RNAi pathway and enter the pathway at the primary miRNA (Pri-miRNA) or mature miRNA stages, respectively. The miRNA mimics/siRNAs lead to the degradation of a complementary mRNA sequence, resulting in the suppression of a cancer-promoting gene. *CRISPR-Cas:* CRISPR-Cas technology can be used to inactive cancer-promoting genes. An expressed nuclease, Cas9, combined with a synthetic small-guide RNA (sgRNA), targets Cas9 to a complementary gene sequence, resulting in DNA cleavage. This cleavage activates error-prone repair, leading to the insertion of deleterious mutations (red insert), thereby inactivating the oncogene. (C) **PRR sensing:** Immunogenic nucleic-acid cargo can stimulate innate immune pathways in immune cells (blue) through pathogen recognition receptors (PRRs). CpG motifs present in pDNA are detected by Toll-like receptor-9 (TLR-9), while intrinsic features of srRNA are detected through TLR-3, -7, or -8, as well as RIG-1. The detection of these pathogen-associated molecular patterns (PAMPs) in pDNA and srRNA activates signaling pathways that induce the production and secretion of IFNs, resulting in immune stimulation in the tumor environment and the generation of an immunologically “hot” tumor. (D) **Cytokines:** Cytokines can be expressed in immune cells (blue: IL-12, IL-7, IL-23, IL-15, OX40L, IL-21, IFN36γ) to remodel the TME by enhancing the infiltration of proinflammatory and cytotoxic immune cells into the tumor environment. Furthermore, these cytokines can improve CPI sensitivity by increasing immune infiltrates into the tumor environment, as well as by upregulating PD-1/PD-L1 factors that can be targeted with CPIs. (E) **Receptors:** T-cell receptors (OX40 or CD137) can be expressed in T-cells (blue) and subsequently activated by antibody agonists (αOX40 or αCD137), resulting in immune activation within the tumor environment. (F) **CAR**: Expression of chimeric antigen receptors (CARs) on the surface of immune cells (blue) allows binding of the CAR to a cancer surface marker (brown), leading to the secretion of cytotoxic and immune stimulation factors (IFN-γ, perforin, granzyme B) and resulting in cancer cell death. (G) **Organ Factory:** Organs (i.e., liver; light red panel) can be used to produce and secrete antibodies or bispecific antibodies (BITES) that enter the circulation, bind a cancer surface marker and an immune cell receptor, and activate cytotoxic immune mechanisms. miRNA, microRNA; siRNA, small interfering RNA; pDNA, plasmid DNA; mRNA, messenger RNA; srRNA, self-replicating RNA; CSE, conserved sequence element; ORF, open reading frame; PolyA, transcription signal; Poly(A), polyadenylation tail; NSP1-4, non-structural protein 1-4; RdRp, RNA-dependent RNA polymerase; TLR, Toll-like receptor; RIG-I, retinoic acid-inducible gene I; IFN, interferon; CPI, check point inhibitor; TME, tumor microenvironment; sgRNA, small-guide RNA.

Standard-of-care treatments for solid cancers include chemotherapy, radiation therapy, and surgery. Recently, the addition of immunotherapy, such as checkpoint inhibitors (CPIs), has transformed some therapeutic outcomes in solid cancers.^21^ However, many late-stage cancers still have poor outcomes and, in some cancers, only 25% of patients are responsive to CPIs.^21^ Genetic medicines use nucleic acids to modify a disease state and offer unique opportunities for precision medicine, with the ability to directly manipulate tumor drivers at a genetic or cellular pathway level. To date, several genetic medicines have been applied using LNPs to treat solid cancers. (Table 1) (Figure 3A: Modalities). These modalities include small non-coding regulator RNAs, such as microRNAs (miRNAs) or small interfering RNAs (siRNAs), or expression of functional proteins using plasmid DNA (pDNA), messenger RNA (mRNA), or self-replicating RNA (srRNA). These modalities can be directed to elicit effects within cancerous cells or non-cancerous support cells in the stroma through gene silencing approaches (CRISPR-Cas or RNAi) or the delivery of toxic genes. Alternatively, payloads can manipulate the immune system by expressing immunomodulators (e.g., cytokines), activating receptors, or expressing immune engagers such as antibodies constructs. (Figure 3). The nucleic acid can also act as stimulator of innate immunity through pathogen recognition receptors (PRRs).

**Table 1:** Nucleic acid LNPs for systemic and intratumoral administration of genetic medicines

Reference	LNP composition	Characterization	Cancer type	Target receptor	Targeting ligand	Modality	Route	Main results	Model
Liu et al.,^22^ 2022	**DAL4:**DOPE:cholesterol:DMG-PEG (20:30:40:0.75)	LNP-DAL: size = 130 nm, ζ = not specified, PDI = 0.05, EE = 90%. TEM = spherical.	Murine melanoma.	None.	None.	IL-12 and IL-27.	i.t.	**B16F10:** reduced tumor growth and survival. In the tumor, increased infiltration of leukocytes, B-cells, CD4^+^ and CD8^+^T cells, NK cells, and macrophages. Increased number of IFN-γ and TNF-α producing immune cells (CD4^+^ CD8^+^T cells, NK cells).	C57BL/6 mice with B16F10 cells.
Li et al.,^23^ 2021	**PL1**:DOPE:cholesterol:DMG-PEG2000 (20:30:40:0.75)	LNP-PL1: size = 167 nm, ζ = +11, PDI = 0.138, EE = 87%. TEM = spherical.	Murine melanoma.	None.	None.	OX40 mRNA.	i.t. (and i.p.)	**B16F10:** reduced tumor growth and increased survival. In the tumor, increased CD8^+^ and CD4^+^ T cells, DCs, and macrophages. When combined with αPD-1/αCTLA4, improved inhibition of tumor growth and increased survival. Inhibition of distal tumors and protection against rechallenge. **A20:** reduced tumor growth and increased survival.	C57BL/6 mice with B16F10 cells. BALB/c mice with A20 cells.
Li et al.,^24^ 2020	**LNP-TT3**:DOPE:cholesterol:C14-PEG2000 (20:30:40:0.75)	size = 100 nm, PI = 0.138, ζ = +4.3, EE = 90%. TEM = spherical.	Murine melanoma.	None.	None.	SrRNA IL-12.	i.t.	**MC-38S B16F10**, **YUMMER1.7, CT26:** reduced tumor growth and increased survival. Increased CD8^+^ T-cell and granulocyte infiltration. **B16F10:** reduced tumor metastases to the lungs. With an αPD1 combination, improved tumor control and survival, and reduced distal tumors.	C57BL/6 mice with B16F10 cells.
Hewitt et al.,^25^ 2020	**Ionizable lipid (not specified)**:DSPC:cholesterol:DMG-PEG2K (50:10:38.5:1.5)	described in M&M (No data). size = 80 to 120 nm in size, ζ = not specified, PDI = not specified, EE = ≥80%.	Murine lymphoma, murine colon adenocarcinoma, murine melanoma.	None.	None.	IL-12 linked to PDGFRbeta transmembrane domain.	i.t.	**MC-38S:** reduced tumor growth and improved survival. **MC-38R:** reduced tumor growth and improved survival. Increased CD4^+^ and CD8^+^ T cells. Reduced CD8^+^/Treg ratio. αPD-L1 combination improved inhibition of tumor growth and survival. Inhibition of distal tumors. **B16-F10-AP3:** reduced tumor growth and improved survival. Increased CD4^+^ and CD8^+^ T cells αPD-L1 combination improved inhibition of tumor growth. **A20:** reduced tumor growth and improved survival.	C57BL/6 mice with MC-38S, MC-38R, B16F10-AP3 cells. BALB/c mice with A20 cells.
Hewitt et al.,^26^ 2019	**Ionizable lipid (Not specified):**DSPC:cholesterol:PEG-lipid (50:10:38.5:1.5)	described in M&M (No data). size = 80–120 nm, ζ = not specified, PDI = not specified, EE > 80%. TEM = spherical.	Murine colon adenocarcinoma, murine melanoma, hepatoma.	None.	None.	IL-23, IL-36γ, and OX40L mRNA.	i.t., s.c., ID	**MC-38:** reduced tumor growth and improved survival. Increased early IL-6, CXCL1, G-CSF and late IFN-γ, TNF-α, IL1-β expression. In the tumor, increased activation markers on NK-T cells and γδ T cells. In the TDLN, increased DC, NK-T and γδ T cells. Inhibition of distal tumors and protection against rechallenge. An αPD1 or αCTL4A combination increased inhibition of tumor growth.	C57BL/6 mice with MC-38 cells.
Hamouda et al.,^27^ 2024	**S-Ac7-DOG**:DSPC:cholesterol:DMG-PEG2000(50:10:38.5:1.5)	size=79.97 nm, ζ= Not specified, PDI = 0.048, EE = 98.50%.	Murine colon carcinoma, mammary adenocarcinoma, melanoma.	None.	None.	IL-21, IL-7, and 4-1BBL mRNA	i.t.	**MC-38: r**educed tumor growth and improved survival. In the tumor, increased total CD8^+^ T cells with granzyme B and IFN-γ infiltration. Increased NK with IFN-γ and granzyme B markers (not total NK cells). In the circulation, increased CD8^+^ T cells. In the TDLN, slight increase in B cells and decreased CD4^+^ T cells. Reduced distal tumors and protection from rechallenge	C57BL/6 mice with MC-38 cells.
Granot-Matok et al.,^28^ 2023	**EA-PIP**:DSPC:cholesterol:DMG-PEG:DSPE-PEG (50:10.5:38:1.4:0.1)	size = 70 nm, ζ = 0 (neutral), PDI = 0.13, EE = 90%.	Murine melanoma.	None.	None.	Pseudomonas exotoxin A mRNA.	i.t.	**B16F10.9:** reduced tumor growth and improved survival with increased Caspase-3 and TUNEL staining in tumors.	C57BL/6 mice with B16F10.9 cells.
Masarwy et al.,^29^ 2025	**L31**:DSPC:cholesterol:DMG-PEG (50:10:38:2)	EGFR-tLNPs: size = 90 nm, ζ = +1, PDI = 0.15, EE = 90%.	Human head and neck squamous carcinoma.	EGFR antibody.	EGFR.	CRISPR/spCas9 mRNA and hSOX2-sgRNA.	i.t.	**UMSCC-104**: reduced tumor growth with enhanced survival and increased Caspase-3 and TUNEL staining.	FOXN1 nude mice with UMSCC-104 cells.
Hsu et al.,^30^ 2013	**DODMA:**EggPC:Chol:PEG-lipid (45:15:35:5)	size = 102.2 nm, ζ = −3.94, PDI = not specified, EE = not specified.	Human hepatocellular carcinoma.	None.	None.	MiRNA-122.	i.t.	**Sk-Hep-1 cells:** inhibition of tumor growth and reduced markers of angiogenesis.	Female athymic mice with Sk-Hep-1 cells.
Wang et al.,^31^ 2024	**XH-07**:cholesterol:DSPC:SINOPEG (40:48:10:2.0)	size = 80 nm, ζ = not specified, PDI = 0.07–0.16, EE = >85%.	Murine colon adenocarcinoma, murine melanoma, murine mammary carcinoma.	None.	None.	SrRNA IL-12.	i.t. and i.v.	**MC-38:** reduced tumor growth and improved survival. **Colon PDX:** reduced tumor growth with increased NK and NK-T infiltration, but reduced CD3^+^ T cells. **B16F10**: combination with αPD-1 reduced tumor growth. Decrease in PMN-MDSCs. **EMT6:** combination with αPD-1 resulted in slight improvement in tumor growth inhibition and survival.	C57BL/6 mice with MC-38 and B16F10 cells. BALB/c mice with EMT6 cells. Human colon PDX in NOG mice.
Wang et al.,^32^ 2024	Cholesterol:DOPE:**DMT7** (25:40:50:2)	size = 70 nm, ζ = 0 (neutral), PDI = <0.2, EE = 95.3%.	Murine melanoma and mammary carcinoma.	None.	None.	IL-12 mRNA.	i.t.	**B16F10:** reduced tumor growth and improved survival. In the tumor, increased CD4^+^ and CD8^+^ T-cells, NK cells, and granulocyte infiltration with increased IL-2, TNF-α, and IFN-γ. In the circulation, increased CD4^+^and CD8^+^ cells with IFN-γ markers. **4T1:** decreased tumor nodes and improved survival. **B16F10** and **4T1:** combination with αPD-1 resulted in improved inhibition of tumor growth, survival. **4T1:** Combination with αPD-1 reduced metastasis.	C57BL/6 mice with B16F10 cells. BALB/c mice with 4T1 cells.
Qin et al.,^33^ 2024	DLin-KC2-DMA:DOPE:cholesterol:C16-PEG2000 Ceramide (*In vitro*: 30:20:49:1 and *In vivo*: 39:10:50:1)	LNP with pDNA: size = 163.0 nm, ζ = +6.63, PDI = 0.22, EE = 91.92%.	Murine melanoma.	None.	None.	pDNA OX40L.	i.t.	**B16F10:** the pDNA or pOX40L inhibited tumor growth and improved survival. Protected mice from rechallenge.	C57BL/6 mice with B16F10 cells.
Yu et al.,^34^ 2025	**LNPLocal lipid**:DSPC:cholesterol:DMG-PEG (46:10:42.4:1.6) **LNPLung lipid:**DSPC:Cholesterol:DMG-PEG (70:4:23.9:2.1)	L**NPLocal**: size = 84.93 nm, PDI = 0.072, ζ = not specified, EE = 94.72%. **LNPLung**: size = 53.23 nm, PDI = 0.131, ζ = not specified, EE = 99.77%.	Murine colon carcinoma, colon adenocarcinoma, and melanoma.	None.	None.	Superagonist IL-15 mRNA.	i.t. (LNPlocal) i.v. (LNPlung)	**CT26, MC-38, B16F10:** reduced tumor growth. **B16F10:** Iin the lung tissue, reduced metastatic burden and CD3^+^ and CD8^+^ cells. In the circulation, increased NK cells.	C57BL/6 mice with B16F10, CT26, and MC-38 cells. BALB/c mice with CT26 cells.
Somu Naidu et al.,^35^ 2025	**Lipid-35**:cholsterol:DSPC:PEG-DMG (40:48.5:10:1.5)	No details provided.	Murine melanoma.	None.	None.	Pseudomonas exotoxin A mRNA.	i.v.	**B16F10.9:** in the lungs, reduced tumor burden and improved survival.	C57BL/6J with B16F10.9 cells.
Lai et al.,^36^ 2018	No details provided	No details provided.	Murine hepatocellular carcinoma.	None.	None.	IL-12 mRNA.	i.v.	**Transgenic HCC model:** reduced tumor growth and survival. In the tumor and TDLN, increased DC and NK-T cells.	Transgenic Tet-inducible hMYC HCC model.
Kang et al.,^37^ 2024	**Ionizable lipid (Not specified)**:DSPC:cholesterol: PEG (42.5:13:43.5:1.0)	No details provided.	Human cervical epidermoid carcinoma.	None.	None.	SiRNA targeted HPV16 E6/E7.	i.v.	**CaSki:** reduced tumor volume. In combination with cisplatin increased inhibition of tumor growth.	Female BALB/c slc-nu/nu mice with CaSki cells.
Quick et al.,^38^ 2022	**MC3**:DSPC:cholesterol:PEG-DMG (50:10:38.5:1.5)	size = 55 nm, ζ = not specified, PDI = 0.092, EE = not specified, TEM = spherical.	Human prostate carcinoma.	None.	None.	SiRNA targeted exon1 of AR.	i.v.	**2Rv1 and LNCaP**: LNP-siARv^m^-LNP inhibited tumor growth with improved survival.	Male NRG mice with 22Rv1 cells.
Rybakova et al.,^39^ 2019	**cKK-E12**:DOPE:cholesterol:C14-PEG2000 (35:16:46.5:2.5)	size = 101 nm, ζ = not specified, PDI = 0.15, EE = 72%.	Breast adenocarcinoma.	None.	None.	Trastuzumab mRNA.	i.v.	**MDA-MB-231-HER**2: inhibited tumor growth.	Female athymic nude mouse with MDA-MB-231-HER2 cells.
Kampel et al.,^40^ 2021	**EA-PIP**:DSPC:cholesterol:DMG-PEG:DSPE (50:10:38:1.5:0.5)	size = 89 nm, ζ = −5.04, PDI = 0.12, EE = not specified.	Human head and neck squamous carcinoma.	EGFR antibody.	EGFR.	siRNA targeted HPV E6 and E7.	i.v.	**UMSCC-104:** reduced tumor growth and improved survival.	FOXN1 nude mice with UMSCC-104 cells.
Sakurai et al.,^41^ 2018	**YSK05**:cholesterol:PEG-DMG (23.3:10:1)	No details provided.	Murine mammary carcinoma.	Cyclic RGD peptide.	αVβ3.	siRNA targeted VEGFR2 and DLL4.	i.v.	**4T1:** reduced VEGFR2 expression in the whole lung	BALB/c mice with 4T1 cells.
Okamoto et al.,^42^ 2018	siRNA:**PP-13** (1:6.8) then add DOPE:cholesterol:DMPG (6:5:2)	Size = 167 nm, ζ = −5.9, PDI = 0.284, EE = not specified.	Breast adenocarcinoma.	HB-EGF Fab'.	HB-EGF.	siRNA targeted PLK1.	i.v.	**MDA-MB-231:** inhibited tumor growth.	BALB/c *nu*/*nu* female mice with MDA-MB-231/mouse cells.
Argueta et al.,^43^ 2024	Undisclosed	No details provided.	Human epithelial breast cancer, human ovarian carcinoma.	No details provided.	None.	CAR TROP2 and gp75 mRNA.	i.v.	**HCC-1954:** inhibited tumor growth. **B16/F10-OVA:** inhibited tumor growth. In the tumor, increased T cells and DC infiltration.	NCG mice with SKOV3, HCC-1954, B16/F10-OVA cells.
Kim et al.,^44^ 2024	**SM-102**:cholesterol:DSPC:DMG-PEG2K:DSPE-PEG2K-Pep (50:38.5:10:1.2:0.3:0.3)	Size = 113 nm, ζ = + >30, PDI = 0.133, EE = 96.4%.	Murine mammary carcinoma.	Peptide.	PD-L1.	PTEN mRNA.	i.v.	**4T1-luc: i**nhibited tumor growth with improved survival.	BALB/c mice with 4T1-luc cells.

(), Molar ratio; CAR, Chimeric antigen receptor; Cas9, CRISPR-associated protein 9; CD4, Cluster of differentiation 4; CD8, Cluster of differentiation 8; CD39, Cluster of differentiation 39 (ectonucleoside triphosphate diphosphohydrolase-1); DC, Dendritic cells; DMG-PE, 1,2-Dimyristoyl-sn-glycero-3-methoxypolyethylene glycol; DOPE, 1,2-Dioleoyl-sn-glycero-3-phosphoethanolamine; DSPC, 1,2-Distearoyl-sn-glycero-3-phosphocholine; EE, Encapsulation efficiency; EGFR, Epidermal growth factor receptor; HB-EGF, Heparin-binding epidermal growth factor-like growth factor; ID, Intradermal; IFN-γ, Interferon gamma; i.t., Intratumoral; i.v., Intravenous; LNP, Lipid nanoparticle; mRNA, Messenger RNA; Myc, MYC proto-oncogene (c-Myc transcription factor); NK, Natural killer cells; nm, Nanometers; PC, 1-Palmitoyl-2-oleoyl-sn-glycero-3-phosphocholine; PD-L1, Programmed death-ligand 1; PDI, Polydispersity index; pDNA, Plasmid DNA; PEG, Polyethylene glycol; PMN-MDSCs, Polymorphonuclear myeloid-derived suppressor cells; PS, 1,2-Dioleoyl-sn-glycero-3-phospho-L-serine; SC, Subcutaneous; SgRNA, Single-guide RNA; SiRNA, Small interfering RNA; SrRNA, Self-replicating RNA; TDLN, Tumor-draining lymph nodes; TNF-α, Tumor necrosis factor alpha; ζ, Zeta potential.

There are two main administration routes of LNPs to deliver genetic medicines: systemic or local. (Figure 1) For local administration, the LNP formulation is directly injected into the tumors. Systemically, the LNPs are generally administered into the bloodstream via intravenous injection. For systemic administration, the LNPs must traffic to the tumors while avoiding uptake in first-pass organs such as the liver, lungs, and spleen, as well as components of the reticuloendothelial system (RES).^45^ Furthermore, LNP delivery can be divided further into either passive or active targeting. (Figure 2) “Passive” delivery relies solely on the LNP formulation chemistry for its biodistribution profile. “Active” delivery requires the conjugation of targeting ligands (e.g., an antibody) to the LNP surface, which can direct the NPs to a cognate receptor.

### Advances in intratumoral delivery of genetic medicines for solid cancers

Direct injection of LNPs with genetic medicine (intratumoral [IT] or intralesional) bypasses the challenges of systemic factors, such as exposure to first-pass organs and the RES. Local administration requires lower amounts of LNP, resulting in high local concentrations, which could enhance anti-tumor effects and reduce dose-limiting toxicity inherent to systemic applications.^46^ IT approaches are most applicable to locally invasive cases or potentially to a limited number of multi-foci metastases that are accessible in organs via guided injections. (Figure 1) However, local administration could also treat advanced disease by generating systemic effects, as demonstrated for immunomodulators that augment CPIs in an adjuvant setting (see **LNP-mRNA delivery as an adjuvant therapy)**, potentially broadening the impact that locally administered LNPs have on disease progression.

### i.t. administration of LNPs with gene silencing modalities

LNP delivery of genetic medicines can inhibit cancer through gene silencing modalities that inactive or suppress cancer drivers. (Figure 3B: **Gene silencing**). CRISPR-Cas functions through a nuclease, Cas9, directed to a target gene by a small guide RNA (sgRNA) that binds a complementary sequence, and Cas9-mediated cleavage results in target gene inactivation by introducing deleterious mutation.^47^ CRISPR-Cas has been used to inactive SRY-Box Transcription Factor 2 (SOX2), a transcription factor overexpressed in cancer and associated with poor survival due to its role in self-renewal and cancer stem cells.^48^ LNPs formulated with Cas9 mRNA and a synthetic sgRNA were tested in a human head-and-neck squamous cell carcinoma (HNSCC) tumor model in FOXN1 nude mice. UMSCC104 tumors treated intratumorally (i.t.) with LNPs packaged with Cas9/SOX2-sgRNA resulted in reduced tumor volume and improved mouse survival.^29^ Other nuclease targets are available and could be applied for local control using CRISPR-Cas technology.^49^

As an alternative gene silencing approach, small regulatory RNAs have also been delivered using LNPs to reintroduce regulation over pro-cancer pathways. miRNAs are small double-stranded RNAs that trigger the endogenous RNAi pathway, resulting in the downregulation of mRNAs with complementary sequences. (Figure 3B: **Gene silencing**) In hepatocellular carcinoma (HCC), the liver-specific miR-122 is lost during oncogenesis, and the reintroduction of a “miRNA mimic” of miR-122 inhibits HCC.^30^ New LNP formulations were developed to improve miRNA-122 delivery when injected i.t. into a Sk-Hep1 HCC model, which reduced tumor growth and suppressed known downstream mRNA targets of miR-122 as well as angiogenesis markers.^30^

Toxins derived from bacteria have been developed for cancer treatment but must be targeted to the tumor as a result of their non-specific toxin mechanisms.^50^ Local delivery using LNPs would restrict the toxin to the tumor site and avoid effects in healthy tissue. LNPs were packaged with mRNA encoding domain III of the pseudomonas exotoxin A (mmPE),^28^ which blocks nicotinamide adenine dinucleotide (NAD^+^)-diphthamide ADP-ribosyltransferase and inactivates eukaryotic translation, resulting in cell death.^51^ LNP-mmPE reduced viability and readily induced apoptosis *in vitro* in B16F10.9 melanoma cells through inhibition of mRNA translation.^28^ In B16F10.9 melanoma tumor-bearing mice, the LNP-mmPE reduced tumor volume, improved mouse survival, and increased the apoptosis marker caspase-3.^28^ Other toxic genes for cancer treatment derived from bacterial and viral sources could also benefit from local delivery using LNPs.^52^^,^^53^

### i.t. administration of LNPs with immune modulators

Unlike factors that directly function on cancer cells, one of the most explored approaches for local LNP delivery to solid cancers is the administration of immune modulators for cancer treatment. In the TME, the immune cells that dictate either pro- or anti-tumor effects represent a complex combination of factors, such as the immune cell population, their phenotypic profiles, and interactions with other cancerous, immune, and non-cancerous cells. Prototypically, an anti-tumor immune population within the tumor environment correlates with the presence of activated, cytotoxic CD8^+^ T cells and NK cells, Th1-oriented CD4^+^ T cells, pro-inflammatory myeloid cells such as DCs and M1-like TAMs, as well as the absence of suppressive cells such as regulatory T cells (Tregs), myeloid-derived suppressor cells (MDSCs), or immunosuppressive M2-like TAMs. (Figure 1) However, it must be noted that TME interactions are complex, and in some cancers the opposite profile can be true, but this is beyond the scope of this review.^54^

Remodeling the TME to be “immune-permissive” with chemokines has a long history (Figure 1). Historically, innate immune activators such as interferon-alpha (IFN-α) were used, but this has expanded to include other IFNs and interleukins (ILs).^55^ Generally, the systemic and local delivery of these factors as recombinant proteins has narrow therapeutic margins, short half-lives, and requires large quantities when administered systemically to achieve sufficient concentrations in the TME for therapeutic benefit. Systemic delivery of immunomodulators is generally limited by unacceptable dose-limiting toxicity. Therefore, local treatment has traditionally been explored using viral vectors expressing immunomodulators, such as adeno-associated viral vectors (AAVs),^56^ adenoviral vectors (Ads),^57^ lentiviral vectors (LVs),^58^ and oncolytic viruses (OVs),^59^ but the sustained expression can result in high serum levels with possible toxicity. Adaptive immune response to viral vectors also prevent repeat dosing, which is often required in aggressive and advanced cancers.^60^ Therefore, the advent of non-viral LNPs represents an immune-compatible format retained in the local TME to transiently express immunomodulators, improving their potency and toxicity profiles.

An immune factor that has been extensively explored is IL-12, which has long been used to induce changes that result in tumor eradication, reduced metastases through anti-angiogenic effects, and elicitation of long-term anti-tumor immunity.^61^ The mechanisms of action are varied but are generally elicited through IL-12 expression from innate immune cells, such as macrophages, monocytes, and DCs, which induce the proliferation of cytotoxic cells (T cells, NK cells, and NK-T cells) and IFN-gamma (IFN-γ) production.^61^ IL-12 has dose-limiting toxicity when administered systemically,^62^ but local expression using LNPs could circumvent this issue.

Early i.t. studies using pDNA to deliver IL-12 with liposomes provided evidence of elevated IFN-γ production in tumors, with reduced tumor proliferation and improved mouse survival in murine colon carcinoma or renal cortical adenocarcinoma models.^63^ Since then, LNPs expressing IL-12 from RNA have shown promising anti-tumor effects, as this format allows for a “burst” of cytokine in the local environment.^22^^,^^24^^,^^25^^,^^31^^,^^32^ LNPs delivering IL-12 have been tested in various cancer models, including melanoma, colon adenocarcinoma, HCC, and mammary cancer murine models. (Table 1) (Figure 3D: **Cytokines**) These studies have demonstrated impressive tumor control and complete remissions, even in challenging models considered highly immunosuppressed. Of note, several studies have shown effects on untreated tumors (distant implanted tumors or metastases), suggesting that broader immunological control can be achieved following local treatment with immunomodulators.^24^^,^^25^

A notable unique feature that can be leveraged with LNPs and RNA to enhance the immunological response is innate immune stimulation. “Immunogenic” LNPs or RNA cargo can complement the effects of IL-12 in the local environment. Ionizable lipids have been identified in LNPs that are capable of eliciting immunogenic cancer cell death (ICD).^24^ (Table 1) ICD is a form of regulated cell death elicited through damage-associated molecular patterns (DAMPs), resulting in cancer cell death and antigen immune presentation.^24^ A novel ionizable lipid (TT3) induced cell death in B16F10 melanoma cell lines.^24^ Furthermore, these immunogenic LNPs were combined with srRNA. (Figure 3A: **Modalities**) Unlike mRNA, which is readily degraded soon after delivery into the cytosol, srRNA increases its copy number by encoding four viral non-structural proteins (NSPs 1–4) that form an RNA polymerase complex (RdRp). The RdRp is recruited to conserved sequence elements (CSEs) to facilitate limited RNA self-replication, which maintains protein expression and extends the kinetics of cytokine expression in the tumor environment.

The srRNA derived from the Venezuelan equine encephalitis virus (VEE) triggers pathogen recognition receptor (PRR) pathways through Toll-like receptors (TLR)-3,-7 and -8, as well as retinoic acid-inducible gene I (RIG-I), resulting in innate IFN production.^24^^,^^31^ (Figure 3C: **PRR sensing**). In the B16F10 melanoma model, i.t. administered LNP-srRNA, without encoding IL-12, reduced tumor growth and increased survival, resulting in increased granulocytes, NK cells, monocytes, and CD4^+^ and CD8^+^ T cells within tumors.^24^ When expressing IL-12 fused to lumican, a collagen-binding protein used to increase local retention of the cytokine, i.t. injection of LNP-srRNA-IL-12 further increased granulocytes and CD8^+^ T cells influx, IFN-γ production, and tumor remissions, as well as mouse survival.^24^ This effect was also observed in other melanoma and colon carcinoma murine models.^24^ To validated these effects in a more representative context of human disease, LNP-srRNA-IL-12 was injected i.t. into a patient-derived xenograft (PDX) tumor derived from a patient with colon cancer in mice that were “humanized” with human peripheral blood mononuclear cells (PMBCs).^31^ Likewise, LNP-srRNA-IL12 inhibited tumor growth with increased NK cell infiltration.^31^

The versatility of LNPs is demonstrated by the ability to co-package IL-12 mRNA with mRNA expressing multiple immune modulatory factors, such as IL-27 and granulocyte-macrophage colony-stimulating factor (GM-CSF) mRNA.^22^ IL-27 is an anti-inflammatory factor involved in improving T cell survival and the generation of a unique memory CD8^+^ T cell population.^64^^,^^65^ GM-CSF induces the differentiation of granulocytes, monocytes, and macrophages in the TME.^66^ New ionizable lipids with di-amino head groups (DAL4) were developed to improve uptake and expression of IL-12 mRNA (Table 1).^22^ When DAL4-LNPs were administered i.t. in a B16F10 melanoma model, the most effective combination was dual mRNAs encoding IL-12 and IL-27 (GM-CSF was less effective), resulting in potent anti-tumor responses that enhanced leukocyte (B cells, T cells, NK cells) and macrophage infiltration, with increased IFN-γ and tumor necrosis factor α (TNF-α)-producing immune cells.

Furthermore, an LNP-IL-12 formulation has been combined with small-molecule drugs, such as a stimulator of interferon genes (STING) agonist.^32^ When treated with a STING agonist, tumor-associated DCs release proinflammatory cytokines and IFNs, which amplify T cell priming and NK cell and DC migration into the tumor.^67^ STING agonists are effective in early disease, but more advanced stages showed lower anti-tumor responses than expected when applied clinically (NCT02675439). T cell exhaustion and deteriorated cytotoxic function may explain this lack of effect, which could be reversed with IL-12. Using LNPs containing an innovative DMT7 ionizable lipid, IL-12 mRNA with MSA-2, a 2^nd^-generation STING agonist, was tested in B16F10 melanoma mice.^32^ MSA-2 alone had limited effects and failed to control tumor growth; however, when combined with i.t. injected LNP-IL-12, significant control of tumor growth and improved mouse survival was observed.^32^ LNP-DMT4-IL-12 alone increased infiltration of CD4^+^ and CD8^+^ T cells and NK cells, but LNP-DMT4-IL-12 combined with MSA-2 further increased CD8^+^ T cells and reduced exhaustion markers.^32^ The most notable effect was observed in the myeloid population, where the combination significantly increased granulocytes as well as levels of anti-tumor cytokines (IL-2, TNF-α, and IFN-γ).^32^

Beyond IL-12, LNP formulations have been used to deliver other factors for immune modulation. In a unique approach, an activating receptor was overexpressed on infiltrating T cells and subsequently activated with an agonist antibody.^23^ (Figure 3E: **Receptors**). The OX40 receptor is involved in stimulating T cells and enhancing tumor-infiltrating lymphocyte (TIL) function.^68^ After screening a series of phospholipid and glycolipid derivatives, “biomimetic” LNPs on T lymphoblasts, Li et al. found a PL1-LNP that effectively delivered mRNA to T cells.^23^ In the B16F10 melanoma model, PL1-LNP OX40 mRNA injected i.t. resulted in OX40 being detected on the surface of tumor-associated CD4^+^ and CD8^+^ T cells, macrophages, and DCs.^23^ When combined with an OX40 antibody agonist, PL1-LNP-OX40 resulted in reduced tumor growth, improved mouse survival, and increased CD8^+^ T cell infiltration.^23^

Alternatively, local expression of the OX40 ligand (OX40L) and pro-inflammatory OX40L-enhancing factors could enhance T cell effector function.^26^(Table 1) (Figure 3D: **cytokines**) IL-23 positively modulates the bridge between innate and adaptive responses^69^ and IL-36γ acts on professional antigen-presenting cells (APCs) and T cells to inhibit tumor growth.^70^ A membrane-anchored version of OX40L packaged into LNPs was injected i.t. into an MC-38 colon adenocarcinoma model.^26^ In the tumor, monocytes, DCs, macrophages, and granulocytes had detectable OX40L, as well as in the tumor-draining lymph nodes (TDLNs).^26^ The treatment was tested in two MC-38 models, which are sensitive (MC-38S) or resistant (MC-38R) to CPI therapy. OX40L alone delayed growth in the sensitive MC-38 model but had no effect on MC-38R tumors.^26^ However, the triple combination of LNPs delivering OX40L, IL-23, and IL-36γ mRNAs significantly improved tumor remissions, even in the immunogenically resistant MC-38R model of colon adenocarcinoma.

LNPs have delivered other cytokine combinations for TME remodeling, namely IL-21, IL-7, and 4-1BB ligands.^27^ (Figure 3D: **cytokines**) IL-21 promotes T cell and NK cell secretion of IFN-γ.^71^ IL-7 induces naive CD8^+^ T cell differentiation into a memory phenotype, protects T cells against adenosine-mediated immunosuppression, and enhances effector functions.^72^ 4-1BB is a member of the tumor necrosis factor (TNF) receptor family and is induced in CD8^+^ T cells following TCR stimulation, with this signaling cascade enhancing proliferation and cytotoxic production.^73^ In an MC-38 colon carcinoma murine model, LNPs packaged with IL-21, IL-7, and 4-1BBL injected i.t. showed increased tumor and serum protein levels. Interestingly, not all combination improved anti-tumor activity. Individually, IL-21 had limited effects on tumor growth, and IL-7 was ineffective.^73^ However, the triplet combination (IL-21, IL-7, and 4-1BB) had pronounced inhibitory effects on tumor growth, with increased CD8^+^ T cells expressing cytotoxic markers (granzyme B and IFN-γ), as well as decreased presence of immune suppressive TAMs in the tumor.^73^ Mechanistically, IL-21 alone increased CD8^+^ T cells expressing cytotoxic granzyme B, while IL-21 and IL-7 were responsible for facilitating T cell infiltration.

As an alternative to mRNA expression formats, pDNA has been used both as an expression template and as an immune stimulator. pDNA is intrinsically immunogenic, harboring CpG motifs that are recognized by TLR9 in APCs, and it can be combined with costimulatory molecules for improved immune effects.^74^ (Figure 3C: **PRR**) Immunogenic pDNA expressing OX40L was injected i.t. in B16F10 melanoma tumors with LNPs and effectively caused complete tumor remissions.^33^

Lastly, and more recently, IL-15 has been used due to its central role in innate and adaptive immunity.^75^ (Figure 3D: **cytokines**) IL-15 superagonists of T cells and NK cells result in therapeutic responses in cancer treatment regardless of dose or route of administration, but toxicity at high doses can result in anorexia, diarrhea, weight loss, and neutropenia due to IL-15-mediated hyperactivation of the immune system.^34^ Using a previously identified LNP for local administration, called LNPlocal, a more potent, structure-optimized IL-15 was delivered i.t. into CT26 colon tumors, showing effective growth inhibition at lower doses with fewer toxic effects observed on mouse weight.^34^

### Systemic delivery of passive LNPs to solid cancers

The i.t. delivery of genetic medicines using LNPs is limited to situations where the tumor lesion is localized (early-stage cancers or limited late-stage cancers) and tumors that are physically accessible by injection. However, late-stage metastatic disease with extensive lesions in tissues or multi-organ involvement could use the systemic administration of LNPs intravenously (i.v.) to passively deliver cargo to the tumor site via the tumor-associated vasculature (Figure 1).

Interestingly, the previously discussed toxic gene, *mmPE*, has also been employed to address lung metastasis in a B16F10.9 melanoma model.^35^ A series of ionized lipids were screened for lung-tropic delivery, and a formulation with lipid 35 demonstrated lung tropism.^35^ The lipid 35-LNPs were packaged with mmPE mRNA, and when delivered systemically into a melanoma model of lung metastasis, lipid35-LNP-mmPE reduced lung weight (i.e., tumor burden) and improved mouse survival. There was a slight increase in liver damage markers in the serum with lipid35-LNP-mmPE, which resolved within 72 h; otherwise, the mice did not show significant adverse effects from the lung-directed LNPs expressing the toxin.^35^

Small regulatory RNAs have also been delivered using LNPs systemically to target human papillomavirus (HPV)-associated cancers. Like miRNA mimics, synthetic siRNAs trigger the endogenous RNAi pathway and can be employed to suppress the HPV oncogenes, E6 and E7. The E6 and E7 oncogenes inactivate or degrade tumor suppressors, and inactivation of the viral oncogenes results in cell death in HPV-associated cancers.^49^^,^^76^ Kang et al. used LNPs loaded with siRNAs targeting HPV E6/E7 genes, which were i.v. administered into tumor-bearing mice implanted with an HPV^+^ cervical cancer in combination with chemotherapy (cisplatin).^37^ Tumor inhibition was noted with either the LNP-siRNA-E6/E7 or cisplatin treatments, but the effects were markedly improved in the combination, with a noted reduction in E6/E7 mRNA levels, reactivation of the tumor suppressor, and induction of cancer cell death.^37^ In another study, LNP-siRNAs have been used to reduce a key cancer driver in prostate cancer: the androgen receptor (AR) splice variants.^38^ The standard treatment for prostate cancer, androgen deprivation therapy, is ineffective in patients with insensitive splice variants that lack the androgen ligand-binding domain (LBD), which maintains cancer growth through androgen-independent transcriptional activation.^77^ In prostate cancer 22Rv1 tumor-bearing mice, which express high levels of the splice variants, tumor growth was slightly but significantly reduced when treated systematically with LNP-siRNA targeting the AR splice variants.^77^

As discussed, immunomodulators have been extensively injected i.t. with LNPs for TME immune restructuring. However, systemic administration of LNPs with cytokines has also been explored for cancer treatment. HCC, which is clinically challenging to treat, was studied by Lai et al., who showed in transgenic HCC murine models that i.v. treatment with LNP-IL-12 reduced HCC tumor growth.^36^ The treatment induced IFN-γ transcript levels in the liver and increased the presence of lymphocytes in the tumor and surrounding liver. In a study using a lung metastasis model of melanoma, a lung-specific LNP formulation (LNPlung) was used to deliver i.v. a high-affinity IL-15, which reduced tumor signal in the lungs.^34^ Histological analysis of the lungs revealed less tumor burden and increased CD8^+^ T cell infiltration.^34^

SrRNA may have the unique feature of higher tumor expression when delivered by LNPs systemically.^31^ An LNP injected i.v. containing srRNA resulted in broad biodistribution in blood and tissue.^31^ However, while most tissues returned to baseline by day 7, expression in the tumor remained 200 times higher, suggesting higher sensitivity to srRNA self-replication.^31^ In a B16F10 melanoma model, a single i.v. injection of LNP-srRNA encoding IL-12 significantly suppressed tumor growth and reduced the presence of immunosuppressive MDSCs, highlighting the potential for systemic administration of srRNA with LNPs for solid cancer treatment.^31^

### Systemically administered targeted LNPs to solid cancers

Although passive targeting has been shown to be feasible for the accumulation of NPs at tumor sites, targeting LNPs to cancer-specific receptors could improve tumor localization after systemic delivery. These “active” or targeted LNPs are generated by the conjugation of targeting ligands to one of the lipid components embedded in the LNP formulation (Figure 2). To date, most genetic medicine studies using targeted LNPs have conjugated whole antibodies or smaller derivatives, such as single-chain variable fragments (ScFv) or fragment antibodies (Fabs). (Table 1) The targeting ligand is generally conjugated to the PEG lipid, which is inserted post-formulation (i.e., the antibody-PEG is mixed with the LNPs); although other methods exist, such as the Anchored Secondary scFv Enabling Targeting (ASSET) system, which uses an E.Coli periplasm anchor embed in the lipid to bind antibodies.^40^ Nevertheless, the ligands direct the LNPs to an intended cell target by binding the surface receptor, after which the LNPs are taken up through receptor-mediated endocytosis.

Epidermal growth factor receptor (EGFR) is often upregulated on the surface of a wide variety of solid cancers, as EGFR signaling promotes cancer progression.^78^ LNPs were targeted to EGFR (αEGFR-LNP) using a well-established anti-EGFR antibody, cetuximab, to direct siRNAs against HPV’s E6 and E7 in HPV^+^ HNSCC.^40^ The αEGFR-LNP-siHPV improved anti-tumor activity, suppressed HPV targets, and induced apoptosis in EGFR-expressing HNSCC cell lines compared to non-targeted LNPs.^40^ When tested in a HPV^+^ SCC104 HNSCC xenograft murine model, i.v. injection of αEGFR-LNP with siRNAs against HPV slightly but significantly inhibited tumor growth more than non-targeted LNPs.^40^ In another study targeting triple-negative breast cancer (TNBC), siRNAs were delivered against the polo-like kinase 1 (*PLK1*) gene, which induces cell death.^42^ A Fab targeting heparin-binding epidermal growth factor-like growth factor (HB-EGF), a common upregulated marker on TNBC, was conjugated to LNPs packaged with siPLK1.^42^ When tested in an MDA-MB-231 tumor model, the HB-EGF-LNP with siPLK1 injected i.v. significantly inhibited tumor growth compared to controls.^42^

Other cell types that indirectly support tumor growth could also be viable targets, such as the tumor-associated vasculature.^41^ Tumor endothelial cells (TECs) support cancer progression in both primary tumors as well as metastatic tumors that have migrated into the lymphatic system. A fusogenic cationic lipid (YSK05) with a cyclic Arginine-Glycine-Aspartic (RGD) peptide, which recognizes αVβ3 integrin—a highly expressed integrin on TECs—was used to direct LNPs with siRNAs targeting vascular endothelial growth factor receptor 2 (VEGFR2) to inhibit angiogenesis.^41^ VEGFR2 is the main receptor for VEGF, which drives tumor-induced angiogenesis.^79^ When delivered systemically in a metastatic BC model of the lungs, the RGD-LNPs with siRNAs against VEGFR2 significantly reduced VEGFR2 expression in the lungs and cancer-affected areas.^41^

As an immune evasion mechanism, tumor cells upregulate suppressive markers that interact with receptors on immune cells to create an immune-tolerate environment. Programmed death ligand 1 (PD-L1) is upregulated on solid tumors and interacts with the programmed cell death protein 1 (PD-1) receptor on immune cells to suppress immune activity.^80^ LNPs targeted to PD-L1 were used to deliver mRNA encoding a tumor suppressor gene, the phosphatase and tensin homolog (PTEN).^44^ When conjugated to a D-peptide (Pep) that binds PD-L1, the targeted Pep-LNPs containing PTEN mRNA were injected i.v. into an aggressive metastatic TNBC model.^80^ Using luciferase-expressing 4T1 cells, the bioluminescent signal in lungs was significantly reduced in the Pep-LNP-PTEN-treated group, with improved mouse survival and effective control of metastasis in the TNBC model.^80^

### *In vivo* LNP delivered CAR-T for solid cancers

As mentioned, CAR-T technology has revolutionized the treatment of some hematological malignancies. Efforts are underway to treat these malignancies by engineering immune cells through the delivery of CAR-Ts via targeted LNPs *in vivo*.^81^^,^^82^ (Figure 3F: **CAR**) Solid cancers are challenging for CAR technology, but LNPs targeted to myeloid cells *in vivo* to express CAR receptors against solid tumor targets have been explored.^43^ Trophoblast cell surface antigen 2 (TROP2) is overexpressed in many epithelial cancers, including breast, lung, colon, ovarian, and pancreatic cancers.^43^ TROP2 CAR mRNA was delivered using a propriety LNP formulation (MT-302) i.v. into non-human primates, and high TROP2 CAR expression was confirmed on CD14^+^ monocytes in the blood.^43^ The i.v.-administered LNP-TROP2-CAR reduced tumor growth in an HCC-1954 BC murine xenograft model.^43^ To further demonstrate the utility of the approach, the myeloid-tropic LNPs were administered i.v. in a B16/F10-OVA melanoma model, which is refractory to CAR or CPI therapy. LNPs packaged with mRNA expressing a CAR targeted to the melanoma marker gp75 showed a robust anti-tumor response, highlighting these LNPs as a promising approach for *in vivo* CAR treatment against solid cancers.^43^

### Passive delivery to tissues as “organ factories”

LNPs can be delivered to tissues *in vivo* to produce antibodies with anti-tumor effects (Figure 3G: **organ factory**). Trastuzumab, which binds to human EGFR 2 (HER2), induces antibody-dependent cellular cytotoxicity in HER2-positive cancers. In BC murine models, i.v. administration of LNPs containing trastuzumab mRNA resulted in its expression and secreation from the liver into the blood stream.^39^ HER2-positive tumors were four times smaller than HER2-negative tumors, with longer morbidity-free survival.^39^ These studies demonstrate how tissues, such as the liver, can be used to express anti-tumor engagers using LNPs.^39^

### LNP delivery of genetic medicines as adjuvant therapy

The PD-L1/PD-1 immunosuppressive axis between tumor and immune cells can be blocked with CPI immunotherapy to improve anti-tumor immune responses, which has revolutionized cancer treatment.^80^ However, many patients remain unresponsive to CPI treatment.^83^ Higher PD-L1 levels in the TME correlate with improved responses to CPIs, along with other factors such as higher T lymphocyte density.^84^ LNPs encoding immunomodulators could improve CPI outcomes by increasing T cell infiltrates and PD-1/PD-L1 levels. (Figure 3D: **cytokines**) This combinatorial effect with CPIs has been observed with immunogenic LNPs and srRNA encoding IL-12 in melanoma,^32^^,^^85^ LNP-IL-12 with STING agonist in melanoma and mammary adenocarcinoma,^32^ OX40 receptor and agonist OX40 antibody in melanoma,^23^ the triple combination IL-23/IL-36γ/OX40 in melanoma or colon adenocarcinoma models,^26^ IL-21/IL-7/4-1BBL combination in mammary adenocarcinoma and melanoma,^27^ and LNP-IL-12 in colon adenocarcinoma.^25^ In two noteworthy studies, IL-12 or the triple combination IL-23/IL-36γ/OX40 were tested with CPIs in challenging models—a CPI-resistant colon adenocarcinoma (MC-38R) or an immunologically “barren” melanoma with minimal infiltrate (B16F10-AP3)—and showed significantly improved anti-tumor effects that were not observed with the CPI or LNP-mRNA individually.^25^^,^^26^

Furthermore, these therapies may generate an effector memory response, which could result in systemic immunity against untreated tumors. This “abscopal” effect has been observed in distal untreated tumors,^25^^,^^26^ in lung metastasis in melanoma models,^23^^,^^85^ and in metastatic models of orthotopic mammary adenocarcinoma.^32^ Although clinical data on systemic anti-cancer immune effects following local tumor treatment are still limited, reports have documented reductions in tumor size in untreated tumors in patients.^86^ Finally, stimulating long-term immune memory could prevent disease recurrence, and protection against cancer rechallenges has been observed in melanoma models following LNP-mRNA monotherapy^26^^,^^27^^,^^33^ or in combination with CPIs.^23^

### Novel LNPs for i.t. delivery: formulations, AI, and beyond

Developing LNP formulations with improved delivery to tumors through i.t. administration could enhance the effectiveness of genetic medicines. Some studies have focused on new ionizable lipids to improve i.t. delivery.^28^^,^^29^^,^^32^^,^^33^^,^^87^ (Table 1) However, improving overall delivery to a tumor when administered i.t. is only one factor affecting the effectiveness of a genetic medicine; understanding the intended cell population within the TME is becoming an important consideration. The LNP dictates the “tropism” in the TME and ultimately the cells expressing the genetic medicine. Despite the importance of understanding LNP tropism, only a few studies have assessed the specific cell types targeted by new LNP formulations when injected i.t. It must be noted that studies covered in this review using systemically administrated LNPs (targeted or passive) have only assessed signal in bulk tumor. Understanding which cell types are most receptive to a specific LNP formulation would influence the resultant effect of the gene therapy and the subsequent therapeutic outcome.

For example, for genetic medicines intended to specifically impact tumor cells (i.e., toxic mRNAs, or siRNAs and CRISPR-Cas directed to an oncogene; Figure 3B: **gene silencing**), maximizing delivery to the tumor is vital. Furthermore, minimizing delivery to the stroma or immune cells would prevent sequestration of the formulation in cells not expected to elicit a therapeutic effect. EA-PIP (lipid 10) LNPs administered i.t. resulted in relatively high tumor-specific delivery (50%–60%) with good specificity for the cancerous cells versus stroma.^28^ Engineering solutions, such as modeling the dispersal pattern of LNPs through multi-needle injection into the lesion,^88^ or using barbed side-hole catheters designed to disperse LNPs along the length of the needle, could also maximize delivery into the tumor.^89^

The rate of dispersal into the tumor has also been explored as a factor to improve delivery. Slow and controlled administration avoids the rapid injection of excessive amounts of LNP, which can result in leakage into the lymphatic system or bloodstream. Prolonged and controlled delivery to enhance tumor accumulation was developed using a micro-syringe chip (MSC) designed for deep tumor penetration and to allow the natural dissolution of LNPs into the local environment.^90^ Novel lipids were designed and optimized for compatibility with the MSC system. The “10PS” lipid showed improved uptake with significantly deeper tumor penetration compared to other formulations.^90^ In a murine 4T1 breast tumor model, the MSC approach showed longer reporter signal durations compared to i.t. injection, peaking at 3–6 h versus 1 h, respectively, demonstrating more sustained delivery to the tumor site.^90^ Although the study used the LNP-MSC system for the delivery of chemical drugs, this type of LNP optimization for sustained and penetrative local delivery of genetic medicines could be explored.

Targeted LNP delivery has mostly been leveraged to improve systemic delivery of LNPs but could also be used to bias LNP cellular uptake when administered i.t., which has been used to improve local uptake with EGFR-targeted LNPs.^29^^,^^40^ These EGFR-targeted LNPs with CRISPR-Cas9 systems to inactive SOX2 showed significantly improved anti-tumor effects compared to non-targeted LNPs.^29^^,^^40^

Likewise, for genetic medicines intended for immune cells, “immune-tropic” LNPs would be beneficial. Only a few studies have elucidated LNP uptake in immune cells when administered i.t.^22^^,^^23^^,^^26^ Importantly, IL-12 and IL-27 may be better expressed from their natural cell population of myeloid cells and DCs.^91^^,^^92^ LNPs with a novel DAL4 ionizable lipid showed high levels of uptake in CD11b^+^ myeloid cell types for expression of the IL-12 and IL-27 cytokines.^22^ However, notably, a substantially higher level of uptake with the DAL4-LNPs was observed in CD19 B cells,^22^ and the DAL4-LNPs could theoretically be used to express or present anti-tumor antibodies in tumor-associated B cells for solid cancer treatment in future studies.^93^ Furthermore, LNPs delivering OX40 receptor mRNA were administered using biomimetic PL1-LNPs that were screened on a T cell line to favor uptake in lymphocytes but actually showed higher uptake in myeloid and DC cell types.^23^ Of note, cytokines expressed from different cell types can have different and even opposite effects on tumor progression (i.e., a cytokine expressed from tumor cells can be pro-tumorigenic compared to the same cytokine expressed from an immune cells), and LNP immune tropism should be better characterized in future studies.

The TME is complex, and other targets in the stroma—such as cancer stem cells, cancer-associated fibroblasts (CAFs), TAMs, MDSC, endothelial cells, pericytes, mesenchymal stromal cells, γδ T cells, tumor-associated neutrophils (TANs), mast cells, eosinophils, or basophils—could be targeted with LNPs for anti-tumor manipulation using genetic medicines. A precise understanding of the unique profile of LNP distribution within the TME could open new genetic medicine targets for cancer treatment.

### Novel advancements in passive and targeted systemic delivery of LNPs

Passive delivery of LNPs to tumors could leverage several advances in LNP development, including, novel lipids (ionizable and PEG), AI-directed selection of lipids, and barcoding of LNPs in high-throughput biodistribution studies. “Barcoding” LNP formulations has been used to improve passive formulations for HNSCC. Sixty-four 7C1-containing LNP formulations with different lipid ratios were packaged with unique DNA oligomers and administered i.v. The isolated tumor contained the most highly represented barcode, identifying an optimal 7C1 LNP formulation for passive HNSCC delivery.^20^ Theoretically, barcodes from passive LNPs could be assessed in sorted cell populations within the tumor to identify cell-type-specific LNP formulations. Furthermore, machine learning (ML) algorithms have been developed to screen a “virtual library” of 40,000 lipids to identify ionizable lipids for improved delivery of mRNA to the lungs,^94^ which could also be leveraged to improve solid tumor delivery formulations. In addition to ionizable lipids, PEG lipids could also be screened for improved “shielding” of LNPs. Using a screening method, new formulations with alternative PEGs were identified with higher tumor-to-liver delivery ratios and were used in HCC models to deliver inhibitory miRNA mimics.^30^

An issue with targeted LNPs is that the conjugation methods used to attach ligands are not flexible for the development of LNPs to new targets. Recent work has highlighted a modulator approach for attaching ligands in order to target LNPs to platelet-endothelial cell adhesion molecule CD31,^95^ vascular cell adhesion molecule 1 (VCAM-1) in brain models,^96^ CD4^+^ T cells,^97^ and CD117^+^ hematopoietic stem cells.^98^ These LNPs could be applicable to solid tumors for targeting cancer-associated vasculature, T cells in the TME, or cancer stems cells, as well as providing a platform method for targeting LNPs to a range of other cancer-associated markers.

### Improving safety for LNP and genetic medicines

Reducing “LNP” leakage and accumulation in first-pass organs (i.e., the liver) could improve the safety of genetic medicines delivered by LNPs by minimizing off-target effects in non-target tissues. Several new LNP formulations have shown improved local accumulation with reduced systemic signal. When compared to LNPs containing the MC3 ionizable lipid, improved local mRNA retention after intramuscular administration was noted with S-Ac7-DOG LNPs, along with reduced liver signal.^87^ However, liver signal was still observed when S-Ac7-DOG LNPs were administrated i.t, suggesting that tumor context will be required to properly assess improved retention with novel formulations.^27^ Alternatively, incorporating tissue-specific regulatory sites into the mRNA can decrease tissue expression. The incorporation of liver-specific miR-122 binding sites into the mRNA has been used to prevent hepatic expression of cytokines when LNPs were injected into tumors.^25^^,^^26^

Lipids can cause reactogenicity, with local inflammation resulting in edema driven largely by macrophage and neutrophil responses to synthetic lipids. A biscarbamate ionizable lipid, S-Ac7-DOG, significantly reduced edema compared to LNPs containing the MC3 ionizable lipid used in commercial drug products.^87^ Furthermore, the PEG lipid in LNPs can induce potent allergic responses, resulting in life-threatening anaphylaxis. New “PEGless” LNPs could resolve the immunogenicity issues associated with PEG,^99^ and also provide new lipid chemistry for the active targeting of LNPs.^100^

### A new dimension in LNP delivery

The “leaky” vasculature concept for NP delivery to solid tumors has been recently challenged, suggesting that the large “gaps” (2000 nm) thought to be present in “leaky” cancer blood vessels are rare and smaller than previously assessed (0.0048% of the blood vessel surface area).^7^ Evidence is emerging for active *trans*-endothelial transport of NPs through transcytosis into the tumor.^7^ A better understanding of this active process could provide new methods for improved delivery of LNPs to tumors.

### Conclusion

Genetic medicines delivered with LNPs have demonstrated utility in solid cancers through effects in the tumor, stroma, and the immune system (Table 1; Figure 3). Clinically, treatments with LNPs containing OX40L/IL-23/IL-36γ (aka mRNA-2752) in combination with CPIs have completed phase 1 trials and achieved the safety and efficacy endpoints.^26^^,^^86^^,^^101^ Furthermore, efforts are underway to deliver CAR technology *in vivo* for the treatment of solid cancers.^81^ (NCT05969041) However, several studies have undisclosed lipid formulations or lack characterization details, and more effort should be made to present formulation details to consolidate and standardize the features required for optimal LNP delivery to solid cancers. (Table 1) Furthermore, the TME of solid tumors presents unique challenges, and a clearer understanding of the mechanism of LNP delivery and LNP tropism within the TME is needed to provide critical insight for improved delivery of genetic medicines. Finally, new methods for designing fit-for-purpose LNPs to penetrate tumors through local or systemic administration could provide effective formulations for delivery of the gene-medicine “toolbox” and unlock the next generation of biomedicines for solid cancers.

## Acknowledgments

This work was supported by the 10.13039/100000002National Institutes of Health, 10.13039/100000054National Cancer Institute, through a P30 CCSG supplement award (NCI, P30CA033572-42S2), and the Elsa U. Pardee Foundation. The content is solely the responsibility of the authors and does not necessarily represent the official views of the National Institutes of Health or the Elsa U. Pardee Foundation.

## Author contributions

F.Y. and T.A.S. contributed to the writing, editing, and figures in the final manuscript.

## Declaration of interests

T.A.S. is a member of the editorial board for Molecular Therapy - Nucleic Acids.

## References

[1] Parhiz H., Atochina-Vasserman E.N., Weissman D. (2024). mRNA-based therapeutics: looking beyond COVID-19 vaccines. Lancet.

[2] Karikó K., Buckstein M., Ni H., Weissman D. (2005). Suppression of RNA recognition by Toll-like receptors: the impact of nucleoside modification and the evolutionary origin of RNA. Immunity.

[3] Karikó K., Muramatsu H., Welsh F.A., Ludwig J., Kato H., Akira S., Weissman D. (2008). Incorporation of pseudouridine into mRNA yields superior nonimmunogenic vector with increased translational capacity and biological stability. Mol. Ther..

[4] Du B., Qin J., Lin B., Zhang J., Li D., Liu M. (2025). CAR-T therapy in solid tumors. Cancer Cell.

[5] de Visser K.E., Joyce J.A. (2023). The evolving tumor microenvironment: From cancer initiation to metastatic outgrowth. Cancer Cell.

[6] Liao D., Johnson R.S. (2007). Hypoxia: a key regulator of angiogenesis in cancer. Cancer Metastasis Rev..

[7] Sindhwani S., Syed A.M., Ngai J., Kingston B.R., Maiorino L., Rothschild J., MacMillan P., Zhang Y., Rajesh N.U., Hoang T. (2020). The entry of nanoparticles into solid tumours. Nat. Mater..

[8] Sauer K., Rakhra K., Wu K., Mehta N.K., Michaelson J.S., Baeuerle P.A. (2024). Intratumoral injection and retention hold promise to improve cytokine therapies for cancer. Front. Oncol..

[9] Wu J. (2021). The Enhanced Permeability and Retention (EPR) Effect: The Significance of the Concept and Methods to Enhance Its Application. J. Pers. Med..

[10] Ngai J., MacMillan P., Kingston B.R., Lin Z.P., Ouyang B., Chan W.C.W. (2023). Delineating the tumour microenvironment response to a lipid nanoparticle formulation. J. Control. Release.

[11] Lin Z.P., Nguyen L.N.M., Ouyang B., MacMillan P., Ngai J., Kingston B.R., Mladjenovic S.M., Chan W.C.W. (2022). Macrophages Actively Transport Nanoparticles in Tumors After Extravasation. ACS Nano.

[12] Chan W.C.W. (2023). Principles of Nanoparticle Delivery to Solid Tumors. BME Front..

[13] Fulton M.D., Najahi-Missaoui W. (2023). Liposomes in Cancer Therapy: How Did We Start and Where Are We Now. Int. J. Mol. Sci..

[14] Shah S., Dhawan V., Holm R., Nagarsenker M.S., Perrie Y. (2020). Liposomes: Advancements and innovation in the manufacturing process. Adv. Drug Deliv. Rev..

[15] Shi L., Zhang J., Zhao M., Tang S., Cheng X., Zhang W., Li W., Liu X., Peng H., Wang Q. (2021). Effects of polyethylene glycol on the surface of nanoparticles for targeted drug delivery. Nanoscale.

[16] Yu H., Dyett B.P., Drummond C.J., Zhai J. (2025). Ionizable Lipid Nanoparticles for mRNA Delivery: Internal Self-Assembled Inverse Mesophase Structure and Endosomal Escape. Acc. Chem. Res..

[17] Hou X., Zaks T., Langer R., Dong Y. (2021). Lipid nanoparticles for mRNA delivery. Nat. Rev. Mater..

[18] Waggoner L.E., Miyasaki K.F., Kwon E.J. (2023). Analysis of PEG-lipid anchor length on lipid nanoparticle pharmacokinetics and activity in a mouse model of traumatic brain injury. Biomater. Sci..

[19] Cheng Q., Wei T., Farbiak L., Johnson L.T., Dilliard S.A., Siegwart D.J. (2020). Selective organ targeting (SORT) nanoparticles for tissue-specific mRNA delivery and CRISPR-Cas gene editing. Nat. Nanotechnol..

[20] Huayamares S.G., Lokugamage M.P., Rab R., Da Silva Sanchez A.J., Kim H., Radmand A., Loughrey D., Lian L., Hou Y., Achyut B.R. (2023). High-throughput screens identify a lipid nanoparticle that preferentially delivers mRNA to human tumors in vivo. J. Control. Release.

[21] Sun Q., Hong Z., Zhang C., Wang L., Han Z., Ma D. (2023). Immune checkpoint therapy for solid tumours: clinical dilemmas and future trends. Signal Transduct. Target. Ther..

[22] Liu J.-Q., Zhang C., Zhang X., Yan J., Zeng C., Talebian F., Lynch K., Zhao W., Hou X., Du S. (2022). Intratumoral delivery of IL-12 and IL-27 mRNA using lipid nanoparticles for cancer immunotherapy. J. Control. Release.

[23] Li W., Zhang X., Zhang C., Yan J., Hou X., Du S., Zeng C., Zhao W., Deng B., McComb D.W. (2021). Biomimetic nanoparticles deliver mRNAs encoding costimulatory receptors and enhance T cell mediated cancer immunotherapy. Nat. Commun..

[24] Li Y., Su Z., Zhao W., Zhang X., Momin N., Zhang C., Wittrup K.D., Dong Y., Irvine D.J., Weiss R. (2020). Multifunctional oncolytic nanoparticles deliver self-replicating IL-12 RNA to eliminate established tumors and prime systemic immunity. Nat. Cancer.

[25] Hewitt S.L., Bailey D., Zielinski J., Apte A., Musenge F., Karp R., Burke S., Garcon F., Mishra A., Gurumurthy S. (2020). Intratumoral IL12 mRNA Therapy Promotes TH1 Transformation of the Tumor Microenvironment. Clin. Cancer Res..

[26] Hewitt S.L., Bai A., Bailey D., Ichikawa K., Zielinski J., Karp R., Apte A., Arnold K., Zacharek S.J., Iliou M.S. (2019). Durable anticancer immunity from intratumoral administration of IL-23, IL-36γ, and OX40L mRNAs. Sci. Transl. Med..

[27] Hamouda A.E.I., Filtjens J., Brabants E., Kancheva D., Debraekeleer A., Brughmans J., Jacobs L., Bardet P.M.R., Knetemann E., Lefesvre P. (2024). Intratumoral delivery of lipid nanoparticle-formulated mRNA encoding IL-21, IL-7, and 4-1BBL induces systemic anti-tumor immunity. Nat. Commun..

[28] Granot-Matok Y., Ezra A., Ramishetti S., Sharma P., Naidu G.S., Benhar I., Peer D. (2023). Lipid nanoparticles-loaded with toxin mRNA represents a new strategy for the treatment of solid tumors. Theranostics.

[29] Masarwy R., Breier D., Stotsky-Oterin L., Ad-El N., Qassem S., Naidu G.S., Aitha A., Ezra A., Goldsmith M., Hazan-Halevy I., Peer D. (2025). Targeted CRISPR/Cas9 Lipid Nanoparticles Elicits Therapeutic Genome Editing in Head and Neck Cancer. Adv. Sci..

[30] Hsu S.-h., Yu B., Wang X., Lu Y., Schmidt C.R., Lee R.J., Lee L.J., Jacob S.T., Ghoshal K. (2013). Cationic lipid nanoparticles for therapeutic delivery of siRNA and miRNA to murine liver tumor. Nanomedicine..

[31] Wang Z., Chen Y., Wu H., Wang M., Mao L., Guo X., Zhu J., Ye Z., Luo X., Yang X. (2024). Intravenous administration of IL-12 encoding self-replicating RNA-lipid nanoparticle complex leads to safe and effective antitumor responses. Sci. Rep..

[32] Wang B., Tang M., Chen Q., Ho W., Teng Y., Xiong X., Jia Z., Li X., Xu X., Zhang X.-Q. (2024). Delivery of mRNA Encoding Interleukin-12 and a Stimulator of Interferon Genes Agonist Potentiates Antitumor Efficacy through Reversing T Cell Exhaustion. ACS Nano.

[33] Qin Y., Rouatbi N., Wang J.T.-W., Baker R., Spicer J., Walters A.A., Al-Jamal K.T. (2024). Plasmid DNA ionisable lipid nanoparticles as non-inert carriers and potent immune activators for cancer immunotherapy. J. Control. Release.

[34] Yu J., Li Q., Zhang C., Wang Q., Luo S., Wang X., Hu R., Cheng Q. (2025). Targeted LNPs deliver IL-15 superagonists mRNA for precision cancer therapy. Biomaterials.

[35] Somu Naidu G., Rampado R., Sharma P., Ezra A., Kundoor G.R., Breier D., Peer D. (2025). Ionizable Lipids with Optimized Linkers Enable Lung-Specific, Lipid Nanoparticle-Mediated mRNA Delivery for Treatment of Metastatic Lung Tumors. ACS Nano.

[36] Lai I., Swaminathan S., Baylot V., Mosley A., Dhanasekaran R., Gabay M., Felsher D.W. (2018). Lipid nanoparticles that deliver IL-12 messenger RNA suppress tumorigenesis in MYC oncogene-driven hepatocellular carcinoma. J. Immunother. Cancer.

[37] Kang S.W., Kang O.J., Lee J.Y., Kim H., Jung H., Kim H., Lee S.W., Kim Y.M., Choi E.K. (2024). Evaluation of the anti-cancer efficacy of lipid nanoparticles containing siRNA against HPV16 E6/E7 combined with cisplatin in a xenograft model of cervical cancer. PLoS One.

[38] Quick J., Santos N.D., Cheng M.H.Y., Chander N., Brimacombe C.A., Kulkarni J., van der Meel R., Tam Y.Y.C., Witzigmann D., Cullis P.R. (2022). Lipid nanoparticles to silence androgen receptor variants for prostate cancer therapy. J. Control. Release.

[39] Rybakova Y., Kowalski P.S., Huang Y., Gonzalez J.T., Heartlein M.W., DeRosa F., Delcassian D., Anderson D.G. (2019). mRNA Delivery for Therapeutic Anti-HER2 Antibody Expression In Vivo. Mol. Ther..

[40] Kampel L., Goldsmith M., Ramishetti S., Veiga N., Rosenblum D., Gutkin A., Chatterjee S., Penn M., Lerman G., Peer D., Muhanna N. (2021). Therapeutic inhibitory RNA in head and neck cancer via functional targeted lipid nanoparticles. J. Control. Release.

[41] Sakurai Y., Hada T., Kato A., Hagino Y., Mizumura W., Harashima H. (2018). Effective Therapy Using a Liposomal siRNA that Targets the Tumor Vasculature in a Model Murine Breast Cancer with Lung Metastasis. Mol. Ther. Oncolytics.

[42] Okamoto A., Asai T., Hirai Y., Shimizu K., Koide H., Minamino T., Oku N. (2018). Systemic Administration of siRNA with Anti-HB-EGF Antibody-Modified Lipid Nanoparticles for the Treatment of Triple-Negative Breast Cancer. Mol. Pharm..

[43] Argueta S., Wang Y., Zhao H., Diwanji N., Gorgievski M., Cochran E., Grudzien-Nogalska E., D'Alessandro J., McCreedy B., Prod'homme T. (2024). In vivo programmed myeloid cells expressing novel chimeric antigen receptors show potent anti-tumor activity in preclinical solid tumor models. Front. Immunol..

[44] Kim Y., Choi J., Kim E.H., Park W., Jang H., Jang Y., Chi S.G., Kweon D.H., Lee K., Kim S.H., Yang Y. (2024). Design of PD-L1-Targeted Lipid Nanoparticles to Turn on PTEN for Efficient Cancer Therapy. Adv. Sci..

[45] Escudé Martinez de Castilla P., Estapé Senti M., Erkens S., van Weerden W.M., Kooijmans S.A.A., Fens M.H., Vader P., Schiffelers R.M. (2025). Reticuloendothelial system blockade does not enhance siRNA-LNP circulation or tumor accumulation in mice. Int. J. Pharm. X.

[46] Moghimi S.M., Simberg D. (2022). Pro-inflammatory concerns with lipid nanoparticles. Mol. Ther..

[47] Cong L., Ran F.A., Cox D., Lin S., Barretto R., Habib N., Hsu P.D., Wu X., Jiang W., Marraffini L.A., Zhang F. (2013). Multiplex Genome Engineering Using CRISPR/Cas Systems. Science.

[48] Mamun M.A., Mannoor K., Cao J., Qadri F., Song X. (2020). SOX2 in cancer stemness: tumor malignancy and therapeutic potentials. J. Mol. Cell Biol..

[49] Scott T.A., Morris K.V. (2021). Designer nucleases to treat malignant cancers driven by viral oncogenes. Virol. J..

[50] Kreitman R.J. (2006). Immunotoxins for targeted cancer therapy. AAPS J..

[51] Li M., Dyda F., Benhar I., Pastan I., Davies D.R. (1996). Crystal structure of the catalytic domain of Pseudomonas exotoxin A complexed with a nicotinamide adenine dinucleotide analog: implications for the activation process and for ADP ribosylation. Proc. Natl. Acad. Sci. USA.

[52] Trivanović D., Pavelić K., Peršurić Ž. (2021). Fighting Cancer with Bacteria and Their Toxins. Int. J. Mol. Sci..

[53] Davis A.M., Scott T.A., Morris K.V. (2022). Harnessing Rift Valley fever virus NSs gene for cancer gene therapy. Cancer Gene Ther..

[54] Giraldo N.A., Sanchez-Salas R., Peske J.D., Vano Y., Becht E., Petitprez F., Validire P., Ingels A., Cathelineau X., Fridman W.H., Sautès-Fridman C. (2019). The clinical role of the TME in solid cancer. Br. J. Cancer.

[55] Zhang Y., Zhang Z. (2020). The history and advances in cancer immunotherapy: understanding the characteristics of tumor-infiltrating immune cells and their therapeutic implications. Cell. Mol. Immunol..

[56] Liu J.Q., Zhu J., Hu A., Zhang A., Yang C., Yu J., Ghoshal K., Basu S., Bai X.F. (2020). Is AAV-delivered IL-27 a potential immunotherapeutic for cancer?. Am. J. Cancer Res..

[57] Tseha S.T. (2022). Role of Adenoviruses in Cancer Therapy. Front. Oncol..

[58] Arce F., Breckpot K., Collins M., Escors D. (2011). Targeting lentiviral vectors for cancer immunotherapy. Curr. Cancer Ther. Rev..

[59] Wang H., Borlongan M., Kaufman H.L., Le U., Nauwynck H.J., Rabkin S.D., Saha D. (2024). Cytokine-armed oncolytic herpes simplex viruses: a game-changer in cancer immunotherapy?. J. Immunother. Cancer.

[60] Shirley J.L., de Jong Y.P., Terhorst C., Herzog R.W. (2020). Immune Responses to Viral Gene Therapy Vectors. Mol. Ther..

[61] Nguyen K.G., Vrabel M.R., Mantooth S.M., Hopkins J.J., Wagner E.S., Gabaldon T.A., Zaharoff D.A. (2020). Localized Interleukin-12 for Cancer Immunotherapy. Front. Immunol..

[62] Wang P., Li X., Wang J., Gao D., Li Y., Li H., Chu Y., Zhang Z., Liu H., Jiang G. (2017). Re-designing Interleukin-12 to enhance its safety and potential as an anti-tumor immunotherapeutic agent. Nat. Commun..

[63] Shi F., Rakhmilevich A.L., Heise C.P., Oshikawa K., Sondel P.M., Yang N.-S., Mahvi D.M. (2002). Intratumoral Injection of Interleukin-12 Plasmid DNA, Either Naked or in Complex with Cationic Lipid, Results in Similar Tumor Regression in a Murine Model. Mol. Cancer Ther..

[64] Salcedo R., Stauffer J.K., Lincoln E., Back T.C., Hixon J.A., Hahn C., Shafer-Weaver K., Malyguine A., Kastelein R., Wigginton J.M. (2004). IL-27 Mediates Complete Regression of Orthotopic Primary and Metastatic Murine Neuroblastoma Tumors: Role for CD8+ T Cells1. J. Immunol..

[65] Liu Z., Liu J.-Q., Talebian F., Wu L.-C., Li S., Bai X.-F. (2013). IL-27 enhances the survival of tumor antigen-specific CD8+ T cells and programs them into IL-10-producing, memory precursor-like effector cells. Eur. J. Immunol..

[66] Armstrong C.A., Botella R., Galloway T.H., Murray N., Kramp J.M., Song I.S., Ansel J.C. (1996). Antitumor Effects of Granulocyte-Macrophage Colony-Stimulating Factor Production by Melanoma Cells. Cancer Res..

[67] Ribeiro A.R.S., Neuper T., Horejs-Hoeck J. (2024). The Role of STING-Mediated Activation of Dendritic Cells in Cancer Immunotherapy. Int. J. Nanomedicine.

[68] Croft M., So T., Duan W., Soroosh P. (2009). The significance of OX40 and OX40L to T-cell biology and immune disease. Immunol. Rev..

[69] Subhadarshani S., Yusuf N., Elmets C.A. (2021). IL-23 and the Tumor Microenvironment. Adv. Exp. Med. Biol..

[70] Wang X., Zhao X., Feng C., Weinstein A., Xia R., Wen W., Lv Q., Zuo S., Tang P., Yang X. (2015). IL-36γ Transforms the Tumor Microenvironment and Promotes Type 1 Lymphocyte-Mediated Antitumor Immune Responses. Cancer Cell.

[71] Isvoranu G., Chiritoiu-Butnaru M. (2024). Therapeutic potential of interleukin-21 in cancer. Front. Immunol..

[72] Wang C., Kong L., Kim S., Lee S., Oh S., Jo S., Jang I., Kim T.D. (2022). The Role of IL-7 and IL-7R in Cancer Pathophysiology and Immunotherapy. Int. J. Mol. Sci..

[73] Kim A.M.J., Nemeth M.R., Lim S.-O. (2022). 4-1BB: A promising target for cancer immunotherapy. Front. Oncol..

[74] Dongye Z., Li J., Wu Y. (2022). Toll-like receptor 9 agonists and combination therapies: strategies to modulate the tumour immune microenvironment for systemic anti-tumour immunity. Br. J. Cancer.

[75] Perera P.Y., Lichy J.H., Waldmann T.A., Perera L.P. (2012). The role of interleukin-15 in inflammation and immune responses to infection: implications for its therapeutic use. Microbes Infect..

[76] Pal A., Kundu R. (2019). Human Papillomavirus E6 and E7: The Cervical Cancer Hallmarks and Targets for Therapy. Front. Microbiol..

[77] Konieczkowski D.J., Otani K., Guan Z., Drumm M.R., Otani Y., Badusi P.O., Chung E.H., Wu S., Davicioni E., Saylor P.J. (2025). Androgen receptor splice variant expression and prostate cancer recurrence after salvage therapy. npj Precis. Oncol..

[78] Laskin J.J., Sandler A.B. (2004). Epidermal growth factor receptor: a promising target in solid tumours. Cancer Treat Rev..

[79] Shah F.H., Nam Y.S., Bang J.Y., Hwang I.S., Kim D.H., Ki M., Lee H.W. (2025). Targeting vascular endothelial growth receptor-2 (VEGFR-2): structural biology, functional insights, and therapeutic resistance. Arch Pharm. Res. (Seoul).

[80] Arafat Hossain M. (2024). A comprehensive review of immune checkpoint inhibitors for cancer treatment. Int. Immunopharmacol..

[81] Bimbo J.F., van Diest E., Murphy D.E., Ashoti A., Evers M.J.W., Narayanavari S.A., Vaz D.P., Rijssemus H., Zotou C., Saber N. (2025). T cell-specific non-viral DNA delivery and in vivo CAR-T generation using targeted lipid nanoparticles. J. Immunother. Cancer.

[82] Hunter T.L., Bao Y., Zhang Y., Matsuda D., Riener R., Wang A., Li J.J., Soldevila F., Chu D.S.H., Nguyen D.P. (2025). In vivo CAR T cell generation to treat cancer and autoimmune disease. Science.

[83] Haddad A.F., Young J.S., Gill S., Aghi M.K. (2022). Resistance to immune checkpoint blockade: Mechanisms, counter-acting approaches, and future directions. Semin. Cancer Biol..

[84] So W.V., Dejardin D., Rossmann E., Charo J. (2023). Predictive biomarkers for PD-1/PD-L1 checkpoint inhibitor response in NSCLC: an analysis of clinical trial and real-world data. J. Immunother. Cancer.

[85] Li N., Li N., Wang Y., Cui N., Wang X., Tang Y., Chu Y., Meng Q., Lin R., Wu Y., Ying B. (2024). Preliminary safety, antitumor activity, and pharmacodynamics of intratumoral ABO2011 (IL-12 mRNA) in patients with advanced solid tumors. J. Clin. Oncol..

[86] Castañón E., Zamarin D., Carneiro B.A., Marron T., Patel S.P., Subbiah V., Mehmi I., Oberoi H.K., El-Khoueiry A., Ridgway B. (2023). Abstract CT004: Intratumoral (IT) MEDI1191 + durvalumab (D): Update on the first-in-human study in advanced solid tumors. Cancer Res..

[87] De Lombaerde E., Chen Y., Ye T., Deckers J., Mencarelli G., De Swarte K., Lauwers H., De Coen R., Kasmi S., Bevers S. (2024). Combinatorial Screening of Biscarbamate Ionizable Lipids Identifies a Low Reactogenicity Lipid for Lipid Nanoparticle mRNA Delivery. Adv. Funct. Mater..

[88] Subbotin V., Fiksel G. (2019). Modeling multi-needle injection into solid tumor. Am. J. Cancer Res..

[89] Pedersoli F., Mohammad I.S., Patel A.K., Kessler J., Chao C., Liu B., Lall C., Guerra C., Park J.J., Boas F.E. (2024). Bioinspired intratumoral infusion port catheter improves local drug delivery in the liver. Sci. Rep..

[90] Kim J., Song S., Gwak M., Cho H., Yun W.S., Hwang N., Kim J., Lee J.S., Kim D.-H., Kim H. (2023). Micro-syringe chip-guided intratumoral administration of lipid nanoparticles for targeted anticancer therapy. Biomater. Res..

[91] Abdalla A.E., Li Q., Xie L., Xie J. (2015). Biology of IL-27 and its role in the host immunity against Mycobacterium tuberculosis. Int. J. Biol. Sci..

[92] Tugues S., Burkhard S.H., Ohs I., Vrohlings M., Nussbaum K., vom Berg J., Kulig P., Becher B. (2015). New insights into IL-12-mediated tumor suppression. Cell Death Differ..

[93] Boucher A., Anderson C., Hinman R., Kindschuh M., Fung J., Wang T., Klooster I., Kim E., Roth C., Vander Oever M. (2025). Engineered human B cells targeting tumor-associated antigens exhibit antigen presentation and antibody-mediated functions. Front. Immunol..

[94] Li B., Raji I.O., Gordon A.G.R., Sun L., Raimondo T.M., Oladimeji F.A., Jiang A.Y., Varley A., Langer R.S., Anderson D.G. (2024). Accelerating ionizable lipid discovery for mRNA delivery using machine learning and combinatorial chemistry. Nat. Mater..

[95] Parhiz H., Shuvaev V.V., Pardi N., Khoshnejad M., Kiseleva R.Y., Brenner J.S., Uhler T., Tuyishime S., Mui B.L., Tam Y.K. (2018). PECAM-1 directed re-targeting of exogenous mRNA providing two orders of magnitude enhancement of vascular delivery and expression in lungs independent of apolipoprotein E-mediated uptake. J. Control. Release.

[96] Marcos-Contreras O.A., Greineder C.F., Kiseleva R.Y., Parhiz H., Walsh L.R., Zuluaga-Ramirez V., Myerson J.W., Hood E.D., Villa C.H., Tombacz I. (2020). Selective targeting of nanomedicine to inflamed cerebral vasculature to enhance the blood-brain barrier. Proc. Natl. Acad. Sci. USA.

[97] Tombácz I., Laczkó D., Shahnawaz H., Muramatsu H., Natesan A., Yadegari A., Papp T.E., Alameh M.G., Shuvaev V., Mui B.L. (2021). Highly efficient CD4+ T cell targeting and genetic recombination using engineered CD4+ cell-homing mRNA-LNPs. Mol. Ther..

[98] Breda L., Papp T.E., Triebwasser M.P., Yadegari A., Fedorky M.T., Tanaka N., Abdulmalik O., Pavani G., Wang Y., Grupp S.A. (2023). In vivo hematopoietic stem cell modification by mRNA delivery. Science.

[99] Xiong S., Liu C. (2025). Breaking the PEG barrier to boost mRNA-LNP therapeutics. Nat. Rev. Mater..

[100] Wang Z., Liu X., Jin J., Liu X., Liu C., Xie F., Wang X., An F., Xiao B., Cheng H. (2026). PEG-Free Monosialoganglioside Lipid Nanoparticles with Spleen-Selective mRNA Delivery for Cancer Immunotherapy. Adv. Funct. Mater..

[101] Ramalingam K., Woody R., Glencer A., Schwartz C.J., Mori H., Wong J., Hirst G., Rosenbluth J., Onishi N., Gibbs J. (2025). Intratumoral Injection of mRNA-2752 and Pembrolizumab for High-Risk Ductal Carcinoma In Situ: A Phase 1 Nonrandomized Clinical Trial. JAMA Oncol..

